# Circadian Effects of Melatonin Receptor-Targeting Molecules In Vitro

**DOI:** 10.3390/ijms252413508

**Published:** 2024-12-17

**Authors:** Kaitlyn Chhe, Maya S. Hegde, Stephanie R. Taylor, Michelle E. Farkas

**Affiliations:** 1 Department of Chemistry, University of Massachusetts Amherst, Amherst, MA 01003, USA; 2 Department of Biochemistry and Molecular Biology, University of Massachusetts Amherst, Amherst, MA 01003, USA; 3 Department of Computer Science, Colby College, Waterville, ME 04901, USA

**Keywords:** bioluminescent reporters, BMAL1, cell culture models, circadian rhythm, melatonin, PER2, period change, phase shift, U2OS cells

## Abstract

Circadian rhythms are important for maintaining homeostasis, from regulating physiological activities (e.g., sleep–wake cycle and cognitive performance) to cellular processes (e.g., cell cycle and DNA damage repair). Melatonin is a key regulator of circadian rhythms and exerts control by binding to melatonin receptor 1 (MT1), decreasing neuronal firing in the suprachiasmatic nucleus (SCN). Previous work studying effects of melatonin on circadian rhythms utilized in vivo models. Since MT1 is also expressed outside of the brain, it is important to study impacts of melatonin on circadian gene oscillations in vitro. We evaluated the effects of melatonin and an MT1 inverse agonist, UCSF7447, in U2OS circadian reporter cell lines, which facilitate detailed assessments of oscillatory changes. We report that cellular circadian rhythms are responsive to treatment with MT1-targeting molecules; their activities are not dependent upon the SCN. Corroborating in vivo data, both melatonin and UCSF7447 lengthened the periods of *BMAL1* and *PER2*, and while melatonin delayed circadian phases, UCSF7447 advanced them. Compounds were also dosed at two different times, however this did not yield changes. Our findings indicate the importance of utilizing in vitro models and that the direct effects of melatonin likely go beyond the SCN and should be explored further.

## 1. Introduction

Circadian rhythms are physical and behavioral changes that follow a cycle of approximately 24 h. They are vital in regulating multiple physical and cellular processes including immunity [1], sleep/wake cycles, metabolism [2], and cell division [3]. The suprachiasmatic nucleus (SCN) is the central pacemaker (“master clock”) that synchronizes these rhythmic processes [4]. In cells, circadian rhythms are controlled by a transcription–translation negative feedback loop. The CLOCK and BMAL proteins activate the transcription of core circadian genes, including periods (PERs) and cryptochromes (CRYs). As PER and CRY accumulate in the cytoplasm, they then translocate into the nucleus to bind to CLOCK and BMAL, thereby inhibiting their own transcription [5].

Melatonin is an important hormone that controls circadian rhythms and sleep. Melatonin is produced by the pineal gland and other tissues in the body and is secreted in a circadian fashion; its levels oscillate in a 24-h pattern and are elevated at night and drop during the day [6]. Melatonin regulates circadian rhythms by reducing neuronal firing of the SCN. This occurs at night via its binding to a G-protein-coupled receptor, melatonin receptor 1 (MT1), to activate G-protein-coupled inwardly rectifying potassium (GIRK) channels [7]. Melatonin can also bind to another G-protein-coupled receptor, melatonin rector 2 (MT2) to inhibit cyclic guanine monophosphate (cGMP) formation and stimulate protein kinase C, a neurotransmitter regulator. Melatonin also has non-receptor-mediated actions that can regulate circadian machinery [8]. Elevated melatonin levels have been found to be associated with proteasome inhibition and enhanced BMAL1 levels, which then in turn affect the levels of PER and CRY, and other clock-controlled genes, including the BMAL1 transcriptional regulators RORs and REV-ERBs [9,10]. Outside of the brain, melatonin can act as an antioxidant, serving as a free radical scavenger [11]. However, it is well established that many cells outside of the SCN also express MT1 and MT2 [12].

Epidemiological studies have suggested that reduced melatonin levels due to performing nightshift work and exposure to light at night may be associated with increased risk of various cancers, including non-Hodgkin’s lymphoma [13], breast cancer [14], colorectal cancer [15], and liver cancer [16], among others. Reduced melatonin levels are also observed in neurological disorders (i.e., Alzheimer’s and Parkinson’s diseases) [17], metabolic diseases (e.g., type 2 diabetes) [18], and liver diseases [16]. MT1, one of the protein receptors that melatonin binds to, has been found in multiple tissues and organs [12] and has itself been implicated in responses to cancers [19]. For instance, when MT1 was overexpressed in breast cancer cell lines, cell proliferation was inhibited and the anti-proliferative effect of exogenous melatonin was amplified, including in in vivo models [20,21].

Given melatonin’s roles in regulating the circadian clock, its effects in disease models [22,23,24], and its widespread use as a sleep aid [25], it is of particular interest to understand the impact(s) of melatonin on circadian rhythms. Previous studies involving exogenous melatonin have found that it can shift circadian phases and re-entrain rhythms in rodents and humans [26,27]. Treatment with melatonin has also resulted in phase advances in mouse SCN neuronal firing in ex vivo studies [28]. Benloucif and Dubocovich demonstrated that mice treated with melatonin at dusk also experienced a phase advance via wheel-running activity, but only when treated at dusk; when melatonin was dosed at dawn, the phase was delayed [29]. Stein et al. also observed similar changes in phase when mice were treated with melatonin at dawn or dusk. They also found that melatonin accelerated re-entrainment in mice [30]. Exogenous melatonin has also been found to be a potential adjuvant in the treatment of diseases such as cancer [31] and neurodegenerative diseases [32].

In melatonin treatment studies performed in cell culture models, the concentrations used were typically determined based on the IC50 value(s) obtained for melatonin, including its effects on cell viability and proliferation. We describe some examples of this work further here, including those that assess effects on clock genes and others that determine changes to cellular behavior and/or characteristics [33,34,35,36]. Studies performed in vitro (treating N2a mouse neuroblasts with 1 µM of melatonin) and ex vivo (treated with 30 µg/kg bodyweight of melatonin) have shown that melatonin results in increased core clock mRNA and protein levels (for *Bmal1*, *Per1*, *Per2*, *Cry1*, and *Cry2*) [37,38]. At the same time, melatonin treatment greater than 100 µM in prostate cancer cells was shown to increase PER2 and CLOCK, as well as reduce BMAL1 levels [33]. Treatment with melatonin at concentrations of or greater than 250 µM has been shown to decrease osteosarcoma (U2OS) cell motility, migration, and invasiveness in vitro [34]. High concentrations of melatonin were also shown to result in anti-proliferative effects in hepatocellular carcinoma-HepG2 cells (treated with 1 mM and 2.5 mM of melatonin) [35] and SKOV3 ovarian cancer cells (treated with 3.4 mM of melatonin) [36]. While the impacts of melatonin on circadian rhythms have been assessed in vivo and ex vivo, and it has shown benefits in in vitro disease models, its effects on oscillations at the cellular level and in non-brain-derived cells have yet to be determined.

Several MT1/2-binding agonists and antagonists have been developed to treat sleep disorders and to aid in the understanding of circadian biology [39,40,41,42,43,44,45,46,47,48,49]. Synthetic derivatives of melatonin, including the MT1/2 agonists, agomelatine [41] and ramelteon [42], possess a greater affinity for the receptors than melatonin itself and advance phases similarly to melatonin [43,44,45,46]. Luzindole is a common MT2-selective antagonist that reduces the phase-shifting effects of melatonin and delays re-entrainment in vivo [47,48]. While antagonists are commonly used in perturbation studies, they typically cannot produce an effect on their own; rather, they block or reverse the effects of an agonist or inverse an agonist [49]. In this work, we were particularly interested in studying whether the effects of an inverse agonist were opposite to those of melatonin in vitro. We did not want to use a compound that negated the effects of melatonin or rendered its receptors inactive. Therefore, we used the inverse agonist UCSF7447. Developed by Stein and coworkers, UCSF7447 is an MT1-targeting molecule that was identified through the docking studies of more than 150 million molecules to an MT1 crystal structure, followed by the synthesis and evaluation of top candidates. UCSF7447 was found to have high affinity and selectivity for both mouse and human MT1 and was shown to decelerate re-entrainment and advance the phase of the mouse circadian clock [30]. Given the potential interest in MT1 as a therapeutic target [50], we hypothesized that if changes to circadian rhythms in cells could be observed following treatment with melatonin, then we could also observe changes with an MT1-binding compound.

In this work, we investigated the effects of melatonin and the MT1-selective inverse agonist UCSF7447 on the oscillations of core clock genes, *BMAL1* and *PER2*, via luciferase-reporters previously generated by our group in U2OS cells [51]. In addition to being a standard in vitro model for circadian rhythms, used widely in studies of the circadian oscillators and their mechanisms [52,53,54], assessment of circadian modulators (including small molecules) [55,56,57] and development of new tools for studying circadian rhythms [58], U2OS cells also express MT1 and MT2 [59]. Compared to using RT-qPCR and Western blot methods used to track core circadian mRNA and protein oscillations, respectively, the reporter approach enables frequent sampling and experiment duration over multiple cycles that lend themselves to detailed analyses [60]. For the purposes of this study, we were particularly interested in the effects of the compounds on period and phase. In terms of circadian oscillations, the period is the length of time from one peak (or trough) to the next peak (or trough). The phase is the time of the peak relative to the dexamethasone pulse, which is used as the reference time (time = 0 h) [61]. Our results demonstrate small but significant and reproducible changes in circadian characteristics for both *BMAL1* and *PER2* following treatment with melatonin or UCSF7447, indicating that SCN-independent effects are discernable. We report, for the first time, that melatonin and UCSF7447 have period effects in vitro. Interestingly, while melatonin delayed *BMAL1* and *PER2* phases, UCSF7447 advanced them, acting as an inverse agonist, as previously described in a report using in vivo models [30]. We have summarized our in vitro and previous in vivo effects on phase in Supporting Information Table S1. In experiments where compound treatments were delayed (by twelve hours), we observed that dosing time did not have a substantial influence on outcomes. Taken together, these studies show that effects of melatonin and other molecules targeting its receptor(s) can be observed in cellular models, which should be part of their evaluations(s) for studies of fundamental biology and therapeutic strategies.

## 2. Results and Discussion

### 2.1. The Effects of Melatonin and a Melatonin Receptor Inverse Agonist on Circadian Oscillations In Vitro

**Figure 1:**
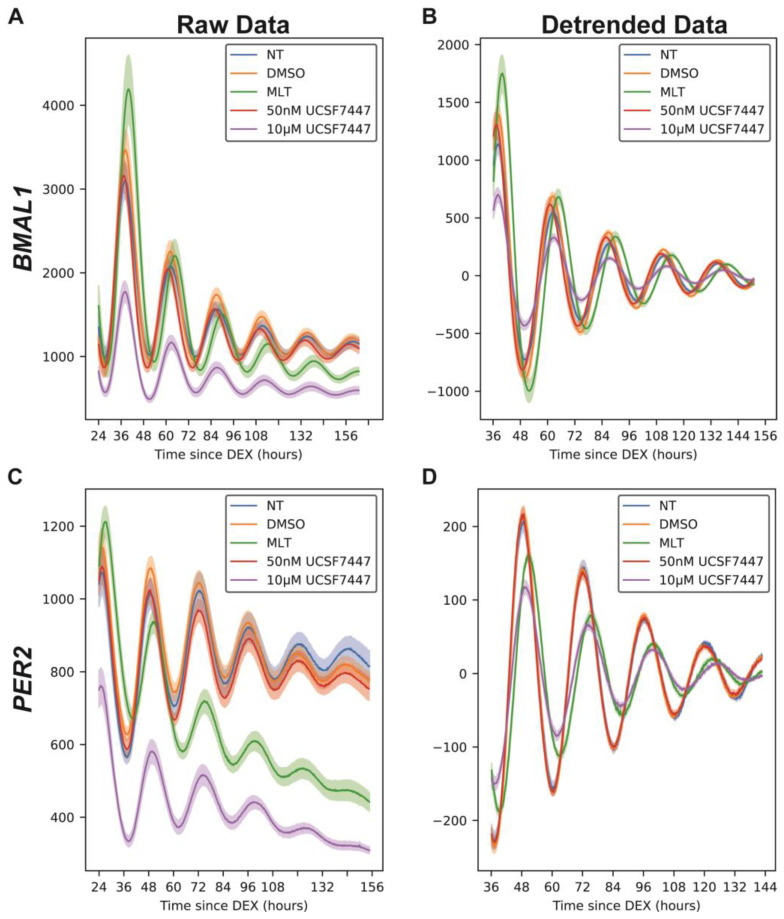
Bioluminescence time-series for *Bmal1:luc* (**A**,**B**) and *Per2:luc* (**C**,**D**). Shown above are raw time-series (**A**,**C**) and time-series after de-trending (**B**,**D**) by removing the average of a 24 h moving window. For each treatment, the mean (raw or de-trended) time-series is plotted as a solid line, with the standard error of the mean as a semi-transparent envelope around it. (For *Bmal1:luc*, N = 10 for NT, N = 11 for DMSO, N = 10 for MLT, N = 11 for 50 nM UCSF7447, and N = 12 for 10 µM UCSF7447. For *Per2:luc* N = 11 for NT, N = 12 for DMSO, N = 11 for MLT, N = 11 for 50 nM UCSF7447, and N = 11 for 10 µM UCSF7447). NT = non-treated, DMSO = dimethyl sulfoxide (vehicle), and MLT = melatonin.

To evaluate the effects of melatonin and the melatonin receptor inverse agonist UCSF7447 on circadian evaluations in vitro, we utilized a standard model, U2OS (bone osteosarcoma) cells, for tracking oscillations. Our lab has previously generated and validated U2OS-based reporter cells for both Bmal1 (U2OS-*Bmal1:luc*) and Per2 (U2OS-*Per2:luc*) [51]. In our initial studies, we treated the cells with melatonin or UCSF7447 using a common approach for dosing and evaluating circadian effects of small molecules, whereupon cells are first synchronized (here with dexamethasone for two hours) and then treated with the compounds, which remain in media with the cells for the duration of the experiment [51,57,62,63,64]. We treated the cells with concentrations of melatonin and UCSF7447 commonly used in various in vitro studies to determine the molecules’ circadian effects in cell culture (1 mM of melatonin and 50 nM and 10 µM of UCSF7447). In preliminary studies to determine what concentration(s) of compounds to use, we did not observe consistent changes to circadian oscillations with lower concentrations of melatonin (0.04 mM–0.5 mM). We are specifically interested in the effects of melatonin independent of the mechanism (whether through the melatonin receptors or via other targets). Dexamethasone pulse, a standard method used to synchronize cells [64,65,66,67], synchronizes U2OS cells by enhancing the expression of *PER1* [68,69]. Bioluminescence intensity was tracked over 7 days. Raw data were de-trended by subtracting a 24 h moving average (Figure 1, Figures S1 and S2).

**Figure 2:**
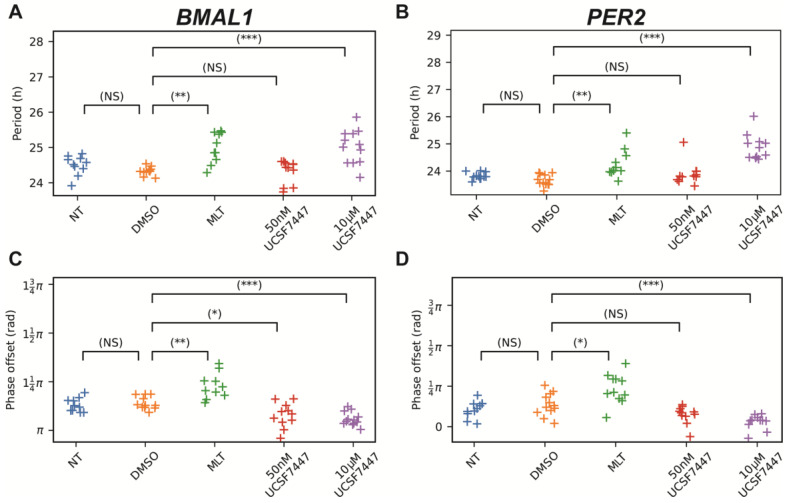
Shown above are the period (**A**,**B**) and phase-offset (**C**,**D**) values estimated by fitting a damped cosine curve to de-trended *Bmal1:luc* (**A**,**C**) and *Per2:luc* (**B**,**D**) time-series. We used a randomization test for differences in means to compare the distributions of measures for non-treated samples to DMSO-treated samples and then to compare DMSO-treated samples to those treated with melatonin and UCSF7447. *p*-values are corrected using the Bonferroni method, and their values are indicated above the bars connecting the pair of treatments being compared (NS indicates “not significant”, * *p* < 0.05, ** *p* < 0.01, and *** *p* < 0.001). NT = non-treated, DMSO = dimethyl sulfoxide (vehicle), and MLT = melatonin.

The results show an anti-phasic relationship in the bioluminescence intensities between *Bmal1* and *Per2*. When *Bmal1* peaks, *Per2* troughs, and vice versa, which is expected since the PER2 complex represses *BMAL1* transcription. Periods and phase offsets were calculated from the de-trended oscillations. We observed that melatonin (MLT) lengthened the period of *Bmal1:luc* by approximately 1 h compared to the dimethyl sulfoxide (DMSO; vehicle) control (Figure 2A). We also found a slight amplitude enhancement, which corresponds with previous findings that demonstrated that MLT enhanced BMAL1 levels [37]. Since BMAL1 is correlated with PER2 expression, we expected and observed a lengthened period for *Per2:luc* as well in Figure 2B. UCSF7447 also lengthened the periods of both *BMAL1* (from 24.33 ± 0.11 h for DMSO to 25.02 ± 0.46 h for UCSF7447) and *PER2* (from 23.66 ± 0.20 h for DMSO to 24.90 ± 0.45 h for UCSF7447) at the higher treatment concentration of 10 µM. It is noteworthy that previous studies did not report period analyses, including effects, following these treatments. Both MLT and 10 µM of UCSF7447 reduced the amplitude of *PER2*.

Additionally, we found that MLT delayed *BMAL1* (by 0.09π rad on average) and *PER2* (by 0.1π rad on average) phases, while UCSF7447 advanced them (by 0.08π rad for *BMAL1* and 0.11π rad for *PER2* on average), all by approximately one hour (Figure 2C,D). These results were confirmed using alternate approximations of phase shift. In these approaches, we detected the peaks (or troughs) of each oscillation and calculated how far ahead of or behind the expected peak (or trough) time was, given the period of oscillation (Supporting Information Figures S3 and S4). A previous study reported that UCSF7447 treatments had effects similar to those of MLT in an in vivo phase-shift model, which was unexpected because UCSF7447 was deemed to be an inverse agonist, including in experiments performed using a re-entrainment model [30]. Interestingly, our phase results confirm UCSF7447’s activity as an inverse agonist, as shown in the re-entrainment model, despite the fact that our experiments are more analogous to those conducted using the phase-shift model. A comparison of the results from the in vitro and in vivo phase-shift experiments is provided in Supporting Information Table S1.

### 2.2. The Effects of Dosing Melatonin and UCSF7447 at a Later Time

**Figure 3:**
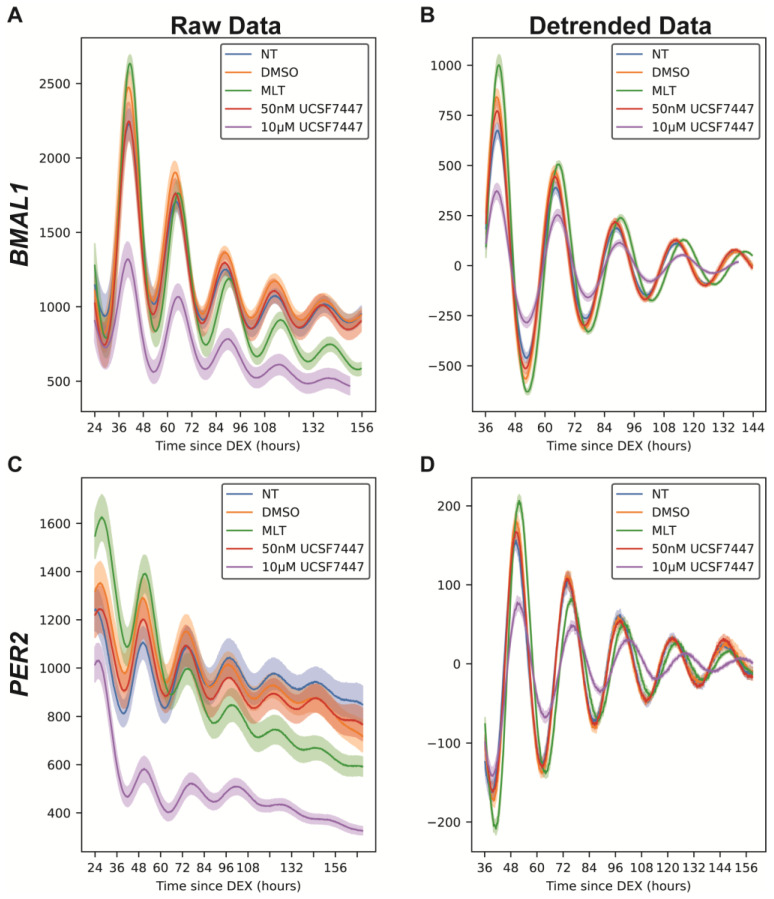
Bioluminescence time-series for *Bmal1:luc* (**A**,**B**) and *Per2:luc* (**B**,**C**) when there is a 12 h delay in treatment after synchronization by dexamethasone. Shown above are raw time-series (**A**,**C**) and time-series after de-trending (**B**,**D**) by removing the average of a 24 h moving window. For each treatment, the mean (raw or de-trended) time-series is plotted as a solid line, with the standard error of the mean as a semi-transparent envelope around it. (For *Bmal1:luc*, N = 11 for NT, N = 11 for DMSO, N = 10 for MLT, N = 9 for 50 nM UCSF7447, and N = 11 for 10 µM UCSF7447. For *Per2:luc*, N = 9 for NT, N = 9 for DMSO, N = 10 for MLT, N = 11 for 50 nM UCSF7447, and N = 11 for 10 µM UCSF7447). NT = non-treated, DMSO = dimethyl sulfoxide (vehicle), and MLT = melatonin.

In this work, we also wanted to determine the impact of dosing time on the periods and phases of *BMAL1* and *PER2* since this can affect the impact and/or efficacy of a compound or treatment [70,71,72,73]. Many non-core clock protein-coding genes are transcribed in a circadian fashion, and their products are targets for drugs [74]. Therefore, chronotherapy (or timing of treatment) is an important factor to consider in drug development. For example, in a clinical study observing the impact of nivolumab dosing on patients with non-small cell lung cancer, the group dosed with nivolumab in the morning had quadruple median progression-free and overall survival numbers compared to the group dosed in the afternoon. Also, nivolumab resistance was mostly observed following afternoon treatment [75]. Stein et al. observed differences in the effects of MLT and UCSF7447 when administration occurred at dusk versus dawn; the phases of the mouse circadian clocks were advanced following MLT or UCSF7447 treatment at dusk, while mice treated with MLT at dawn experienced a phase delay (those treated with UCSF7447 at dawn did not experience phase changes) [30]. For our delayed dosing experiments, U2OS-*Bmal1:luc* and U2OS-*Per2:luc* were treated with MLT or UCSF7447 12 h after dexamethasone synchronization (versus immediately following synchronization, utilized above). De-trended data were generated by subtracting a 24 h moving average from the raw data (Figure 3, Figures S5 and S6).

**Figure 4:**
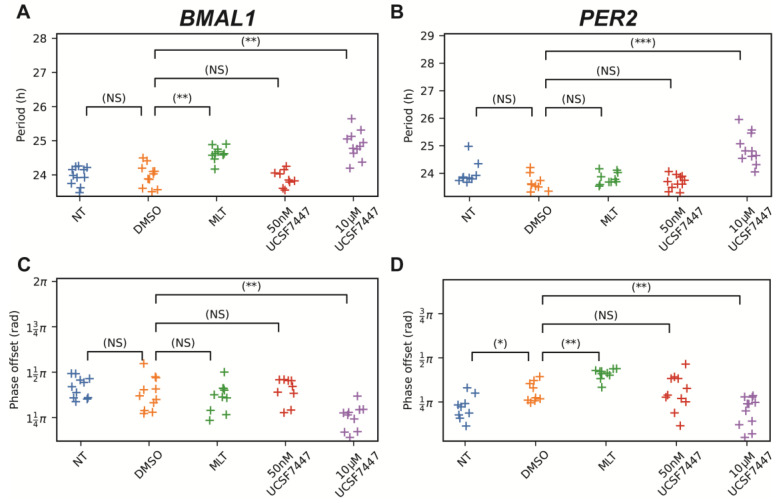
Shown above are the period (**A**,**B**) and phase-offset (**C**,**D**) values estimated by fitting a damped cosine curve to de-trended *Bmal1:luc* (**A**,**C**) and *Per2:luc* (**B**,**D**) time-series for which the treatments were delayed by 12 h after synchronization by dexamethasone. We used a randomization test for difference in means to compare the distribution of measures for non-treated samples to DMSO-treated samples and then to compare DMSO-treated samples to those treated by melatonin and UCSF7447. *p*-values are corrected using the Bonferroni method, and their values are indicated above the bars connecting the pair of treatments being compared (NS indicates “not significant”, * *p* < 0.05, ** *p* < 0.01, and *** *p* < 0.001). NT = non-treated, DMSO = dimethyl sulfoxide (vehicle), and MLT = melatonin.

As expected, *BMAL1* and *PER2* promoter activity were largely anti-phasic. MLT and UCSF7447 lengthened the periods of *BMAL1* in cells dosed 12 h after synchronization (23.98 ± 0.31 h for DMSO, 24.63 ± 0.20 h for MLT, and 24.88 ± 0.39 h for UCSF7447; Figure 4A), aligning with the results from immediate treatment. Only UCSF7447 significantly lengthened the period of *PER2* (23.65 ± 0.28 h for DMSO and 24.90 ± 0.54 h for UCSF7447; Figure 4B), whereas in the immediate treatment, both MLT and UCSF7447 lengthened the period of *PER2*. We also observed similar changes in *BMAL1* amplitudes in the MLT and 10 μM UCSF7447 treatments across both dosing regimens. Specifically, MLT slightly enhances amplitude, while the 10 μM UCSF7447 reduces amplitude. For *PER2*, MLT seems to increase the amplitude, which did not occur in the immediate dosing, and 10 μM of UCSF7447 decreases the amplitude as observed for treatment following synchronization.

Additionally, we found that MLT delayed *PER2* phases (by 0.1π rad on average), which was in agreement with the results from the immediate treatment. However, MLT did not delay the phase of *BMAL1* when treatment was delayed, as it did when the treatment was immediate. UCSF7447 advanced the phases of both *Bmal1:luc* and *Per2:luc* (by 0.13π rad for *Bmal1* and 0.11π rad for *Per2*), which is also similar to treatment following synchronization. We note that since 0.08 π is approximately one hour, significant shifts were observed. Although in vivo experiments showed that the dosing time of MLT impacted the circadian phase (causing delays at dawn and advances at dusk) and that phase shift due to UCSF7447 was unexpectedly similar to that of MLT, in vitro experiments showed no effect of dosing time and showed the expected opposite effects of MLT and UCSF7447 on phase (one delayed and the other advanced, respectively). Delaying the treatment did not reveal phase-dependent effects of either MLT or UCSF7447 treatment. Instead, it showed diminished effects. This could be caused by individual oscillators drifting out of phase with each other, and as a result, each oscillator would respond differently to the dosing and therefore would reduce the sizes of phase shifts in particular.

## 3. Materials and Methods

### 3.1. Cell Culture

The cell lines used in this work were established previously by the Farkas Lab [51]. U2OS-*Bmal1:luc* and U2OS-*Per2:luc* cells were cultured in high glucose, no glutamine Dulbecco’s modified eagle medium (DMEM, Gibco, Waltham, MA, USA) supplemented with 10% fetal bovine serum (Corning, Corning, NY, USA), 2 mM of L-glutamine (Gibco), 100 U/mL penicillin–streptomycin (Gibco), 1× non-essential amino acids (Cytiva, Marlborough, MA, USA), and 1 mM of sodium pyruvate (Gibco).

### 3.2. Cell Synchronization and Bioluminescence Recording

Cells were plated in 35 mm dishes at a density of 4 × 10^5^ cells per dish and synchronized when 90% confluent. Each experiment had 6 replicates per condition (N = 6). The experiments were repeated twice for a total of 12 replicates per condition. For synchronization, cells were treated with 100 nM of dexamethasone (Sigma-Aldrich, St. Louis, MO, USA) diluted in cell culture media and incubated for 2 h at 37 °C in a 5% CO_2_ atmosphere. After synchronization, cells were washed with phosphate-buffered saline (PBS) and then treated with 2 mL of luminometry recording media containing DMSO (vehicle control, 0.2%, MP Biomedicals, Santa Ana, CA, USA), melatonin (1 mM, TCI America, Portland, OR, USA), or UCSF 7447 inverse agonist (10 μM or 50 nM, Sigma-Aldrich). Luminometry recording media comprised 11.25 mg/mL powdered DMEM (Sigma-Aldrich), 4 mM of sodium bicarbonate (Fisher Scientific, Waltham, MA, USA), 5% fetal bovine serum (Corning), 10 mM of HEPES (HyClone, Logan, UT, USA), 100 U/mL penicillin–streptomycin (Gibco), and 0.5 mM of D-luciferin (Thermo Scientific, Waltham, MA, USA) dissolved in autoclaved Millipore water. After dosing, the cells were transferred to a LumiCycle luminometer (Actimetrics, Wilmette, IL, USA) for data collection for 7 days at 37 °C.

For the delayed treatments, synchronized cells were cultured in U2OS growth media for 12 h (37 °C in 5% CO_2_ atmosphere) and then dosed with 2 mL of melatonin, UCSF7447, or vehicle control in recording media. After treatment, the cells were transferred to a LumiCycle luminometer for data collection for 7 days at 37 °C.

### 3.3. Data Analysis

Each time-series was pre-processed to exclude the initial 24 h transient, then de-trended by removing a 24 h moving average. To estimate circadian period and phase, the de-trended time-series was fit (scipy.optimize.differential_evolution from Scipy v. 1.13.1) to a damped cosine curve with a linear baseline ($Ae^{-\lambda t}\cos\left(\frac{2\pi }{\tau }-\theta \right)+c_{0}+c_{1}t$, where t is time in hours; τ is the period in hours, and θ is the phase in radians) [76]. A time-series was considered an outlier if the fit was poor (the coefficient of determination between the data and the fit was less than 0.9) or if its period or phase was more than 2 standard deviations from the mean of all time-series for the same reporter and treatment condition. The circular mean and standard deviation measures were used for the phase (scipy.stats.circmean and scipy.stats.circstd) [76].

A two-sided permutation test was employed to determine whether the mean period or phase differed between treatment conditions (scipy.stats.permutation_test with 10,000 re-samples) [76]. *p*-values were then adjusted using the Bonferroni correction to account for multiple comparisons.

## 4. Conclusions

Melatonin is an important hormone that regulates the physiology and the behavior of organisms. Due to melatonin’s pro-apoptotic, anti-proliferative, and anti-angiogenic effects, the use of melatonin as a pharmacological agent is of great interest. With the addition of MT1/2 as drug targets to treat various diseases (such as sleep disorders, depressive disorders, and some cancer types), understanding the effects of melatonin and MT1/2-targeting molecules in vivo and in vitro is important. Previous in vitro and ex vivo studies were largely performed using neuronal cells or the SCN. However, melatonin can also function and bind to the MT1/2 receptors externally from the SCN. Therefore, we deemed assessments of melatonin outside of the SCN (and in vitro) to be necessary.

In our work, we explored the effects of melatonin and an MT1-targeting molecule in vitro using U2OS cells, a well-established and often-utilized cell culture model of circadian rhythms. We observed the effects of MLT and an MT1 inverse agonist, UCSF7447, on the promoter activity of *BMAL1* and *PER2* at two different dosing times in vitro. We report that the periods of *BMAL1* and *PER2* are lengthened when cells are treated immediately following synchronization. To the best of our knowledge, this is the first demonstration of period alteration due to melatonin or an MT1 targeting molecule. We also observed that UCSF7447 behaves as an inverse agonist in terms of phase; MLT delays the phases of *BMAL1* and *PER2*, while UCSF7447 advances the phases. Interestingly, our phase results correspond with those results obtained using an in vivo model of re-entrainment, which demonstrated that UCSF7447 acts as an inverse agonist. This is in contrast with findings from an in vivo model for phase shift, which unexpectedly found the opposite (despite our in vitro model being a closer approximation to the phase-shift model). Although in vivo studies demonstrated that melatonin induces or advances a phase delay, depending on the time of day it is administered, our in vitro work showed no time-dependent effects.

Taken together, our results indicate that melatonin and MT1-targeting agents have demonstratable effects on circadian rhythms independent of the SCN. Our results show that melatonin and UCSF7447 have both acute phase-shifting and persistent period-altering capabilities. This is especially striking for UCSF7447, which both advances the phase and slows down the oscillations. Given their physiological and pharmacological importance, it is surmised that the brain-independent roles of melatonin and MT1 should be studied further. Further, this study elucidates the merit and worthiness of performing evaluations of melatonin and any related therapeutics in vitro as an alternative or at least complement to animal studies.

## Data Availability

The original data presented in this study are openly available via the Data Repository at ScholarWorks, The URL is https://hdl.handle.net/20.500.14394/55085, (accessed on 5 December 2024).

## Supplementary Materials

The following supporting information can be downloaded at: https://www.mdpi.com/article/10.3390/ijms252413508/s1.
