# Supplementary material for: Circadian Effects of Melatonin Receptor-Targeting Molecules In Vitro
Source: Int J Mol Sci. 2024 Dec 17;25(24):13508. doi: 10.3390/ijms252413508 (PMC11727910; doi:10.3390/ijms252413508)
Supplement: Supplementary file 1 [file ijms-25-13508-s001.zip › ijms-3300344-supplementary.pdf]

Supplementary Materials

Circadian Effects of Melatonin Receptor-Targeting Molecules In Vitro

tlyn Chhe 1, Maya S. Hegde 2, Stephanie R. Taylor 3,* and Michelle E. Farkas 1,*

[1] Department of Chemistry, University of Massachusetts Amherst, Amherst, MA 01003, USA

[2] Department of Biochemistry and Molecular Biology, University of Massachusetts Amherst, Amherst, MA 01003, USA

[3] Department of Computer Science, Colby College, Waterville, ME 04901, USA

Additional Data Analysis Methods

To provide a second set of estimates for circadian shifts, we implemented a method that uses the peaks and troughs of the approximately 24h oscillations in the de-trended timeseries as markers. Scipy.signal.find_peaks (scipy version 1.13.1) was used to locate peaks that were at least 18 hours apart [1]. To ensure that the first peak was not associated with the transient, we only considered peaks occurring after t=36h for *Per2-luc* recordings and t=48h for *Bmal1-luc* recordings. We then used the same function to find troughs by reversing the signs of the data. We discarded any troughs that occurred before the first peak. To estimate the shift, we compared the timing of each peak and each trough to its expected time for an idealized curve. The ideal *Per2-luc* recording begins with a peak at t=0, with all subsequent peaks following at τ h intervals (where τ, tau, is the period estimated from the damp-cosine fitting described in the main text). The ideal *Bmal-luc* recording begins with a trough at t=0 and all subsequent troughs follow at τ-h intervals. We report the differences between the actual and expected times for each peak and each trough. If a treatment condition results in a shift, then we expect to see a similar peak-time difference across all peaks and troughs for that treatment, and expect that the changes between controls and treatments will be statistically significant changes. Even if the untreated condition is not ideal, if a treatment results in differences relative to the untreated condition, then we have observed a phase shift.

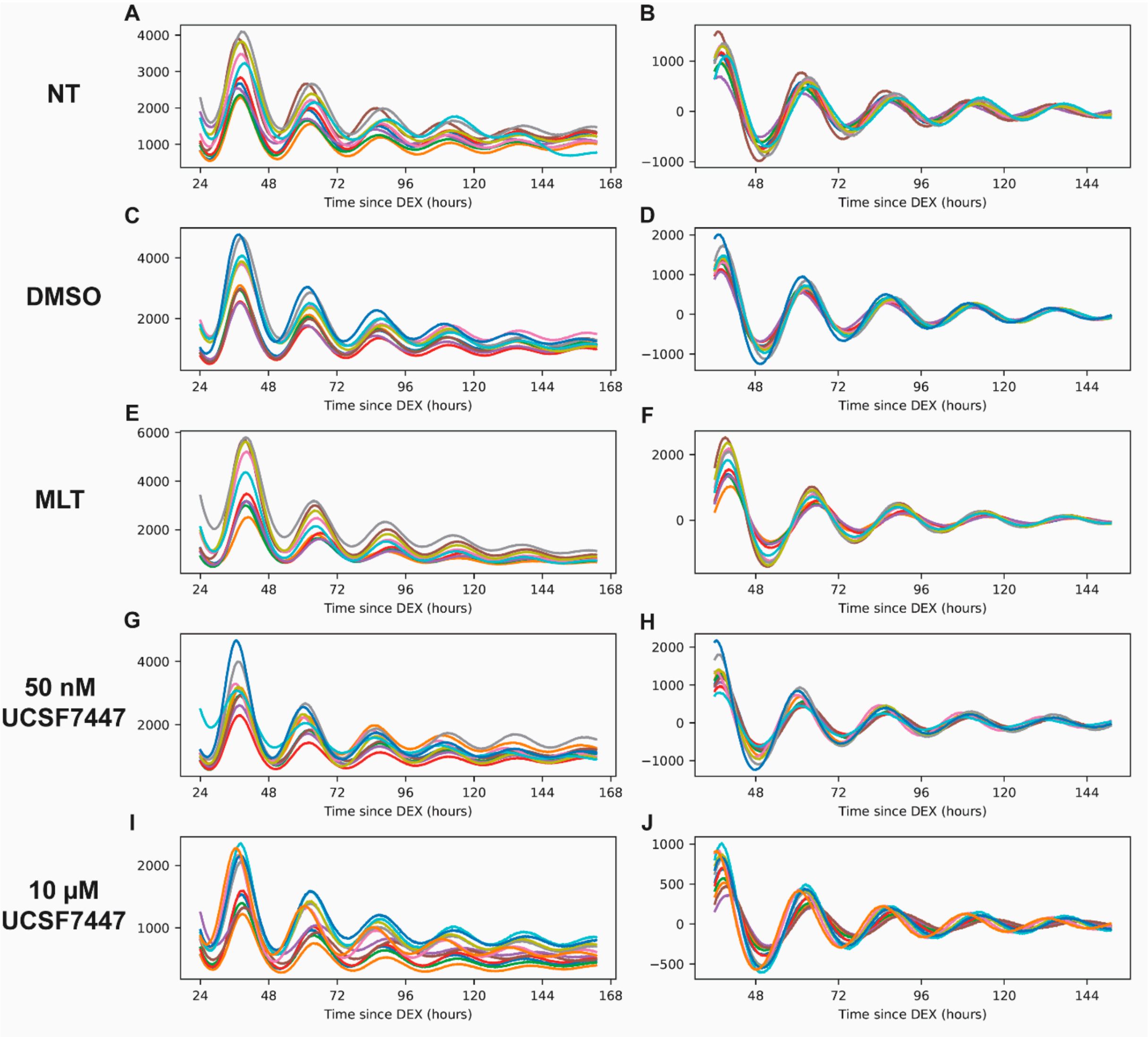

Figure S1. Shown are the individual *Bmal1-luc* times series for results shown in **Figure 1** with a 24-h transient removed (A, C, E, G, I) and after removing the trend from the data by subtracting the mean of a 24-h sliding window (B, D, F, H, J). Each row of sub-figures represents a different treatment. NT = non-treated, DMSO = dimethylsulfoxide (vehicle), MLT = melatonin.

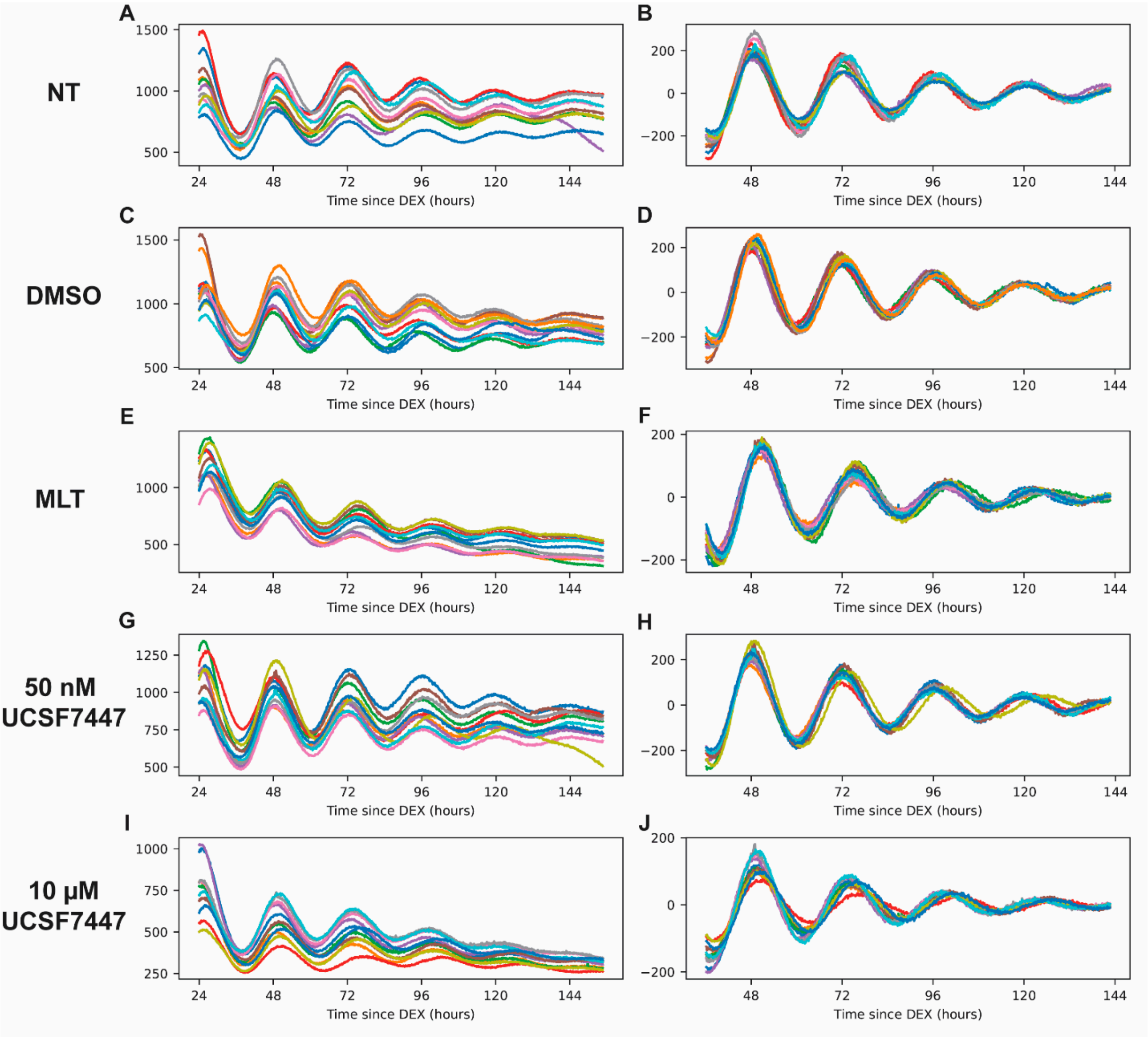

Figure S2. Shown are the individual *Per2-luc* times-series for results shown in **Figure 1** with a 24-h transient removed (A, C, E, G, I) and after removing the trend from the data by subtracting the mean of a 24-h sliding window (B, D, F, H, J). Each row of subfigures represents a different treatment. NT = non-treated, DMSO = dimethylsulfoxide (vehicle), MLT = melatonin.

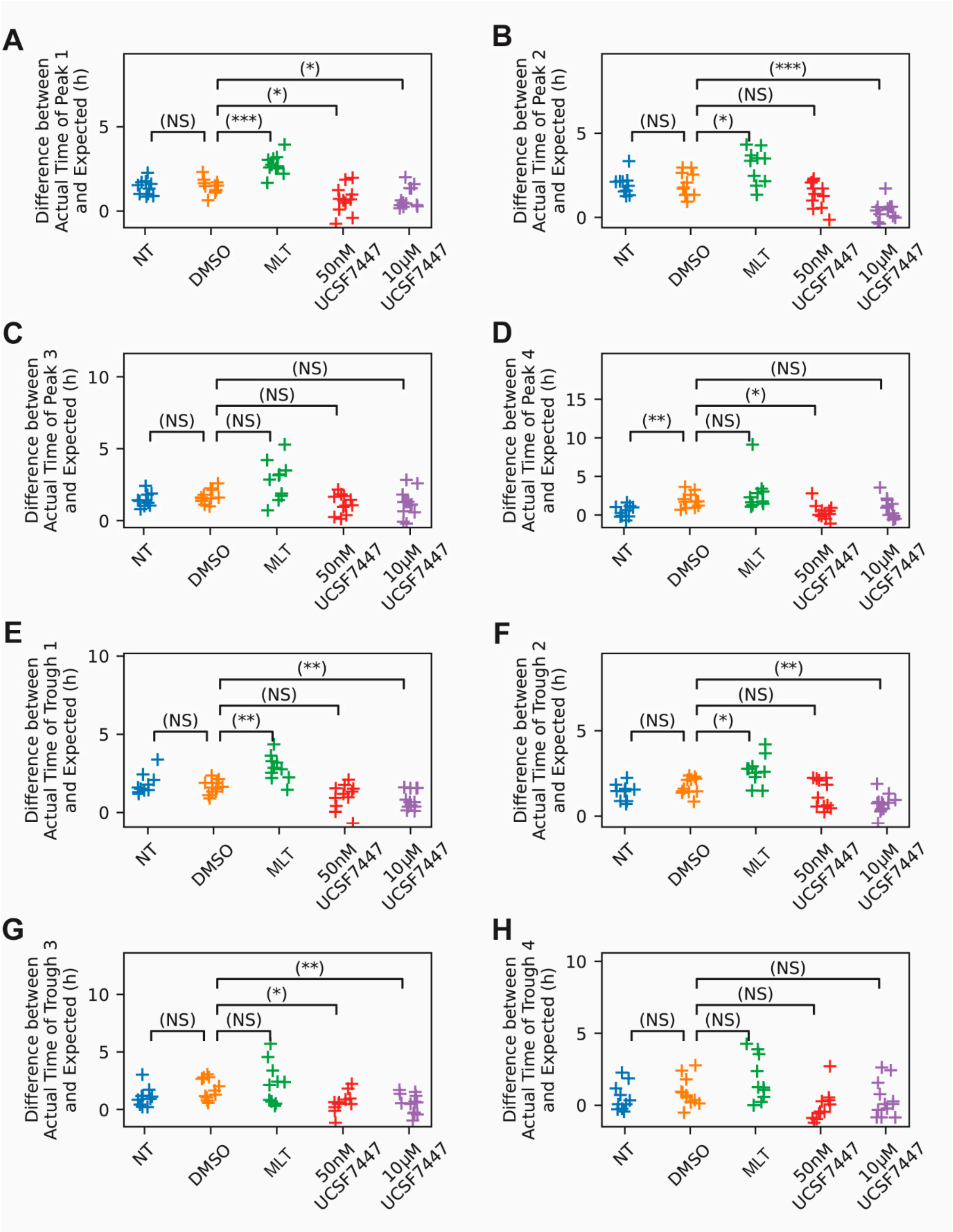

Figure S3. Shown are the phase offsets of *Bmal1-luc* time-series as estimated by each of the first four peaks (A-D) and troughs (E-H) starting 36 hours after dexamethasone treatment. Each phase offset is calculated as the actual time of peak/trough minus the predicted time, assuming the rhythm begins with a trough at time t=0 using the period estimated from fitting the damped sine curve. We use a randomization test for difference in means to compare the distribution of measures for non-treated samples to DMSO treated samples and then to compare DMSO treated samples to those treated with melatonin and UCSF7447. P-values are corrected using the Bonferroni method and their values are indicated above the bars connecting the pair of treatments being compared (NS indicates "not significant," * $p < 0.05$, ** $p < 0.01$, *** $p < 0.001$). NT = non-treated, DMSO = dimethylsulfoxide (vehicle), MLT = melatonin.

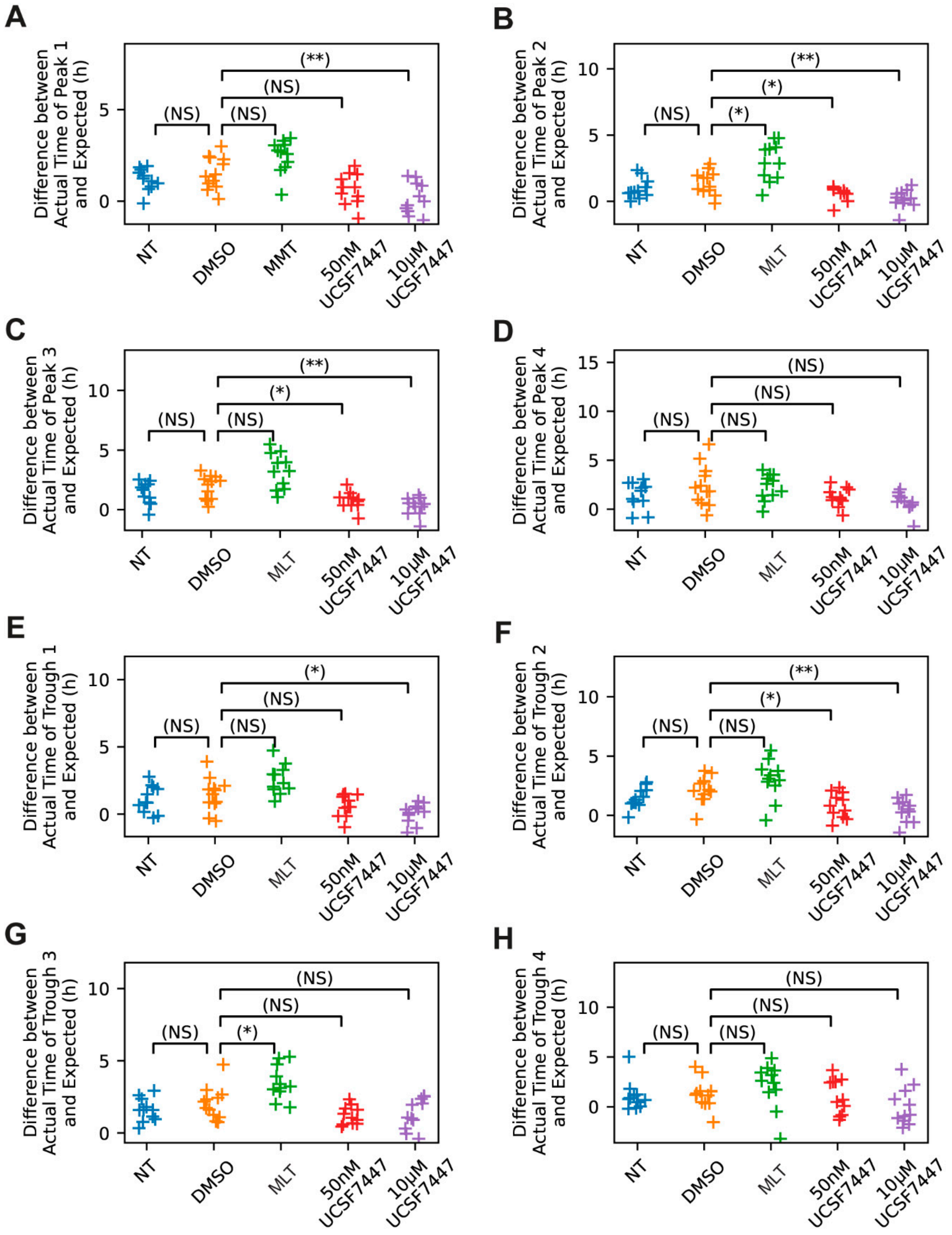

Figure S4. Shown are the phase offsets of *Per2-luc* time-series as estimated by each of the first four peaks (A-D) and troughs (E-H) starting 36 hours after dexamethasone treatment. Each phase offset is calculated as the actual time of peak/trough minus the predicted time, assuming the rhythm begins with a peak at time t=0 using the period estimated from fitting the damped sine curve. We use a randomization test for difference in means to compare the distribution of measures for non-treated samples to DMSO treated samples and then to compare DMSO treated samples to those treated with melatonin and UCSF7447. P-values are corrected using the Bonferroni method and their values are indicated above the bars connecting the pair of treatments being compared (NS indicates "not significant," $*p < 0.05$, $**p < 0.01$, $***\ p < 0.001$). NT = non-treated, DMSO = dimethylsulfoxide (vehicle), MLT = melatonin.

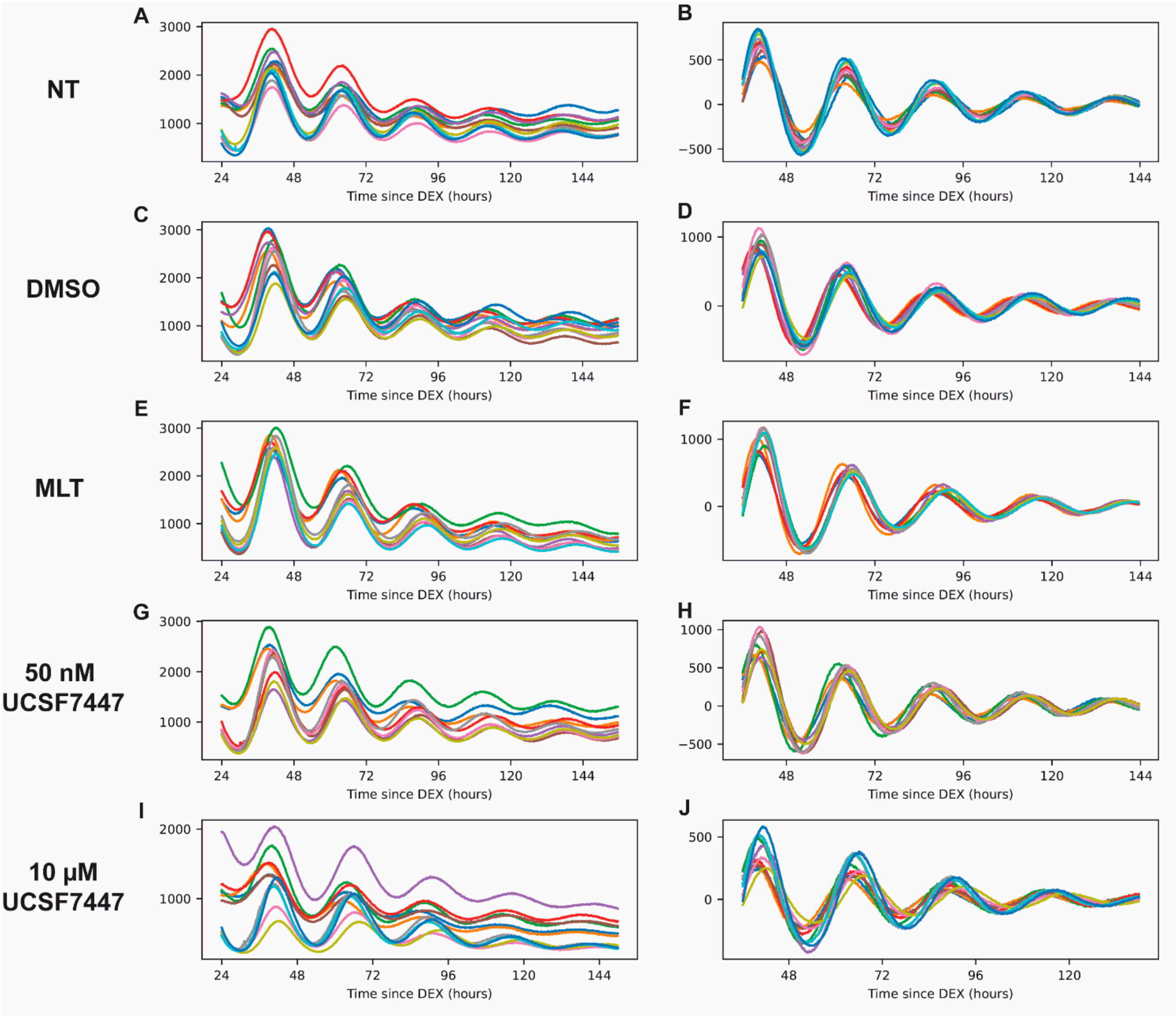

Figure S5. Shown are the individual delayed-treatment *Bmal1-luc* times series for results shown in **Figure 3** with a 24-h transient removed (A, C, E, G, I) and after removing the trend from the data by subtracting the mean of a 24-h sliding window (B, D, F, H, J). Each row of subfigures represents a different treatment. NT = non-treated, DMSO = dimethylsulfoxide (vehicle), MLT = melatonin.

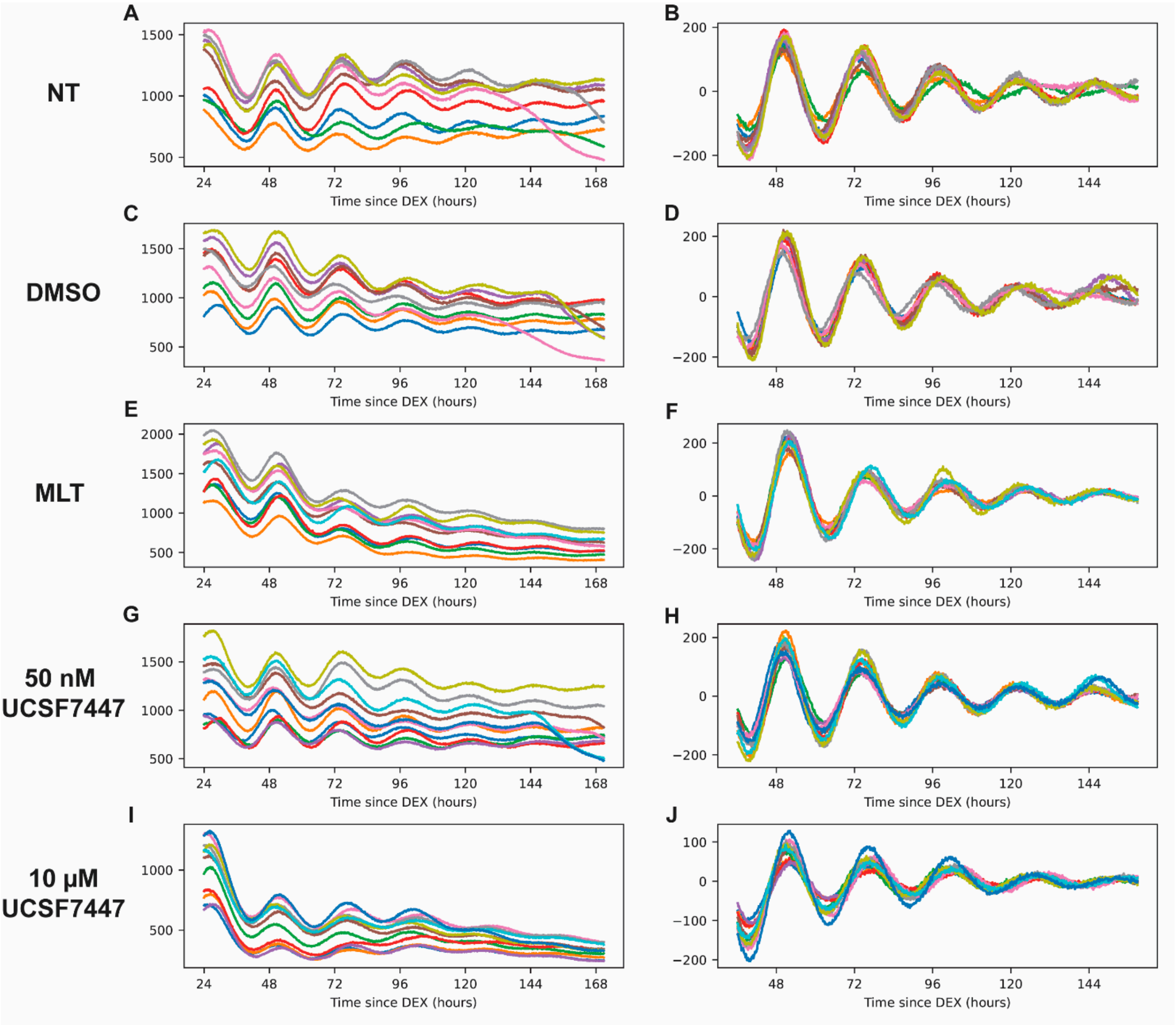

Figure S6. Shown are the individual delayed-treatment *Per2-luc* times-series for results shown in **Figure 3** with a 24-h transient removed (A, C, E, G, I) and after removing the trend from the data by subtracting the mean of a 24-h sliding window (B, D, F, H, J). Each row of subfigures represents a different treatment. NT = non-treated, DMSO = dimethylsulfoxide (vehicle), MLT = melatonin.

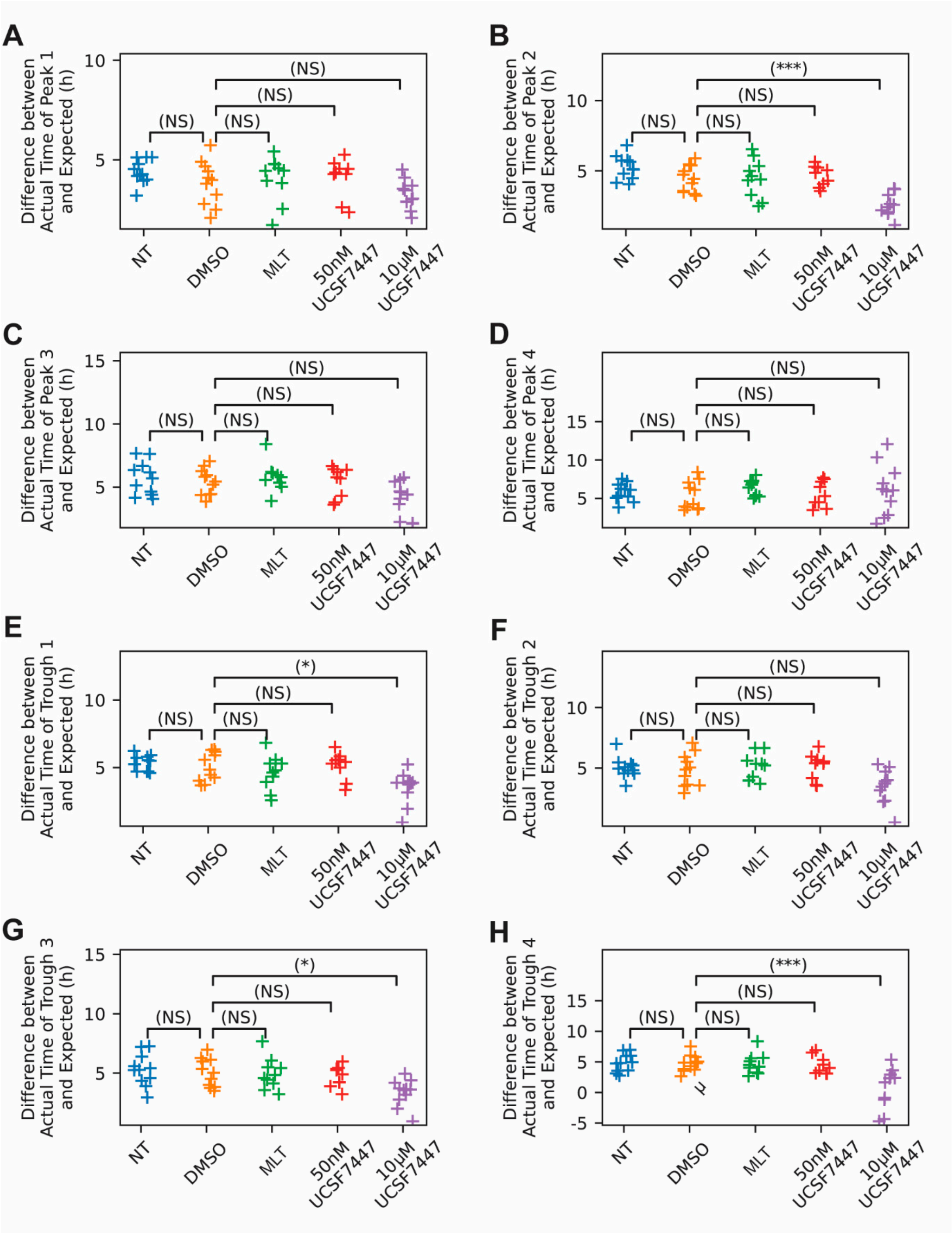

Figure S7. Shown are the phase offsets of delayed-treatment *Bmal1-luc* time-series as estimated by each of the first four peaks (A-D) and troughs (E-H) starting 36 hours after dexamethasone treatment. Each phase offset is calculated as the actual time of peak/trough minus the predicted time, assuming the rhythm begins with a trough at time t=0 using the period estimated from fitting the damped sine curve. We use a randomization test for difference in means to compare the distribution of measures for non-treated samples to DMSO treated samples and then to compare DMSO treated samples to those treated with melatonin and UCSF7447. P-values are corrected using the Bonferroni method and their values are indicated above the bars connecting the pair of treatments being compared (NS indicates "not significant," *p < 0.05, **p < 0.01, *** p < 0.001). NT = non-treated, DMSO = dimethylsulfoxide (vehicle), MLT = melatonin.

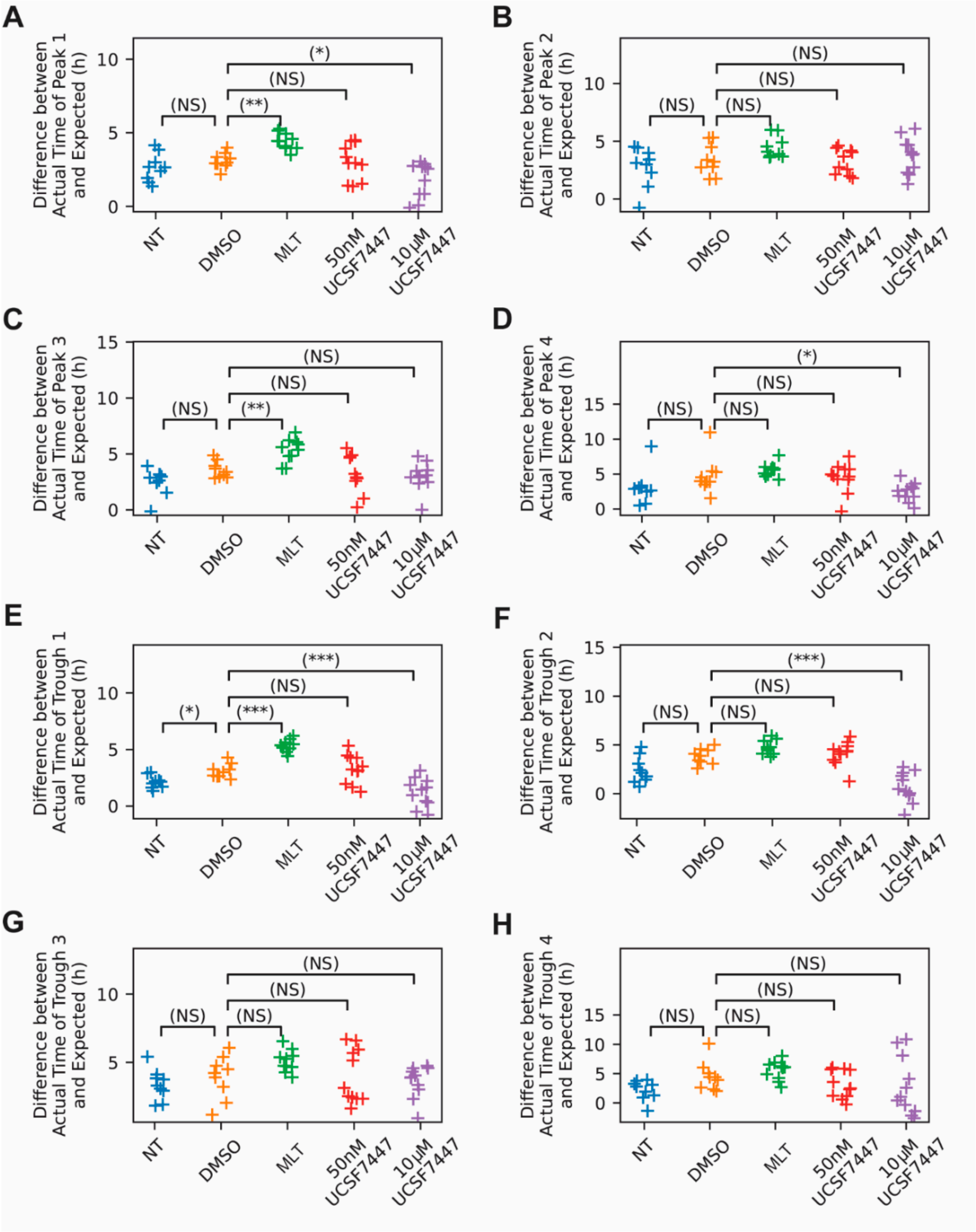

Figure S8. Shown are the phase offsets of delayed-treatment *Per2-luc* time-series as estimated by each of the first four peaks (A-D) and troughs (E-H) starting 36 hours after dexamethasone treatment. Each phase offset is calculated as the actual time of peak/trough minus the predicted time, assuming the rhythm begins with a peak at time t=0 using the period estimated from fitting the damped sine curve. We use a randomization test for difference in means to compare the distribution of measures for non-treated samples to DMSO treated samples and then to compare DMSO treated samples to those treated with melatonin and UCSF7447. P-values are corrected using the Bonferroni method and their values are indicated above the bars connecting the pair of treatments being compared (NS indicates “not significant,” *p < 0.05, **p < 0.01, *** p < 0.001). NT = non-treated, DMSO = dimethylsulfoxide (vehicle), MLT = melatonin.

Compound	Model	Summary of Phase Effects
Melatonin	*in vivo*	Treatment at dusk resulted in a phase advance; treatment at dawn resulted in a phase delay [2,3].
	in vitro	Treatment immediately after synchronization resulted in delayed BMAL1 and PER2 phase. Delayed treatment resulted in PER2 phase delay, but no effect on BMAL1 phase.
UCSF7447	*in vivo*	Mice treated at dusk had a slight phase advance; treatment at dawn did not affect the phase [3].
	in vitro	Treatment immediately after synchronization resulted in BMAL1 and PER2 phase advances. Delayed treatment also led to phase advances of BMAL1 and PER2.

Table S1. Summary of effects observed following treatment with melatonin or UCSF7447. *In vivo* results are from cited papers utilizing phase-shift models. *In vitro* results are from data presented here.

References

1. Virtanen, P.; Gommers, R.; Oliphant, T.E.; Haberland, M.; Reddy, T.; Cournapeau, D.; Burovski, E.; Peterson, P.; Weckesser, W.; Bright, J.; et al. SciPy 1.0: Fundamental Algorithms for Scientific Computing in Python. *Nat Methods* **2020**, *17*, 261–272.
2. Benloucif, S.; Dubocovich, M.L. Melatonin and Light Induce Phase Shifts of Circadian Activity Rhythms in C3H/HeN Mouse. *J. Biol. Rhythms* **1996**, *11*, 113-125.
3. Stein, R.M.; Kang, H.J.; McCorvy, J.D.; Glatfelter, G.C.; Jones, A.J.; Che, T.; Slocum, S.; Huang, X.-P.; Savych, O.; Moroz, Y.S.; et al. Virtual Discovery of Melatonin Receptor Ligands to Modulate Circadian Rhythms. *Nature* **2020**, *579*, 609–614.
